# *Toxoplasma gondii* prevalent in China induce weaker apoptosis of neural stem cells C17.2 via endoplasmic reticulum stress (ERS) signaling pathways

**DOI:** 10.1186/s13071-015-0670-3

**Published:** 2015-02-04

**Authors:** Jie Zhou, Xiaofeng Gan, Yongzhong Wang, Xian Zhang, Xiaojuan Ding, Lingzhi Chen, Jian Du, Qingli Luo, Teng Wang, Jilong Shen, Li Yu

**Affiliations:** Department of Microbiology and Parasitology, Anhui Provincial Laboratory of Microbiology and Parasitology, Anhui Key Laboratory of Zoonoses, Anhui Medical University, Hefei, 230032 PR China; Clinical Laboratory, People’s Hospital of Huaibei, Huaibei, 235000 PR China; College of Life Sciences, Zhejiang Sci-Tech University, Hangzhou, 310018 PR China; School of Life Sciences, Anhui University, Hefei, 230039 PR China; Department of Biochemistry, Anhui Medical University, Hefei, 230032 PR China; HTS & Compound Management, HD Biosciences Corporation, Shanghai, 201201 PR China

**Keywords:** Toxoplasma gondii, TgCtwh3, C17.2, Apoptosis

## Abstract

**Background:**

*Toxoplasma gondii*, an obligate intracellular pathogen, has a strong affinity for the nervous system. TgCtwh3, a representative Chinese 1 *Toxoplasma* strain prevalent in China, has the polymorphic features of the effectors ROP16_I/III_ with type I and GRA15_II_ with type II *Toxoplasma* strains. The interaction of this atypical strain with host cells remains extremely elusive.

**Methods:**

Using a transwell system, neural stem cells C17.2 were co-cultured with the tachyzoites of TgCtwh3 or standard type I RH strain. The apoptosis levels of C17.2 cells and the expression levels of related proteins in the endoplasmic reticulum stress (ERS)-mediated pathway were detected by flow cytometry and Western blotting.

**Results:**

The apoptosis level of C17.2 cells co-cultured with TgCtwh3 had a significant increase compared to the negative control group; however, the apoptosis level in the TgCtwh3 group was significantly lower than that in the RH co-culture group. Western blotting analyses reveal that, after the C17.2 cells were co-cultured with TgCtwh3 and RH tachyzoites, the expression levels of caspase-12, CHOP and p-JNK in the cells increased significantly when compared to the control groups. After the pretreatment of Z-ATAD-FMK, an inhibitor of caspase-12, the apoptosis level of the C17.2 cells co-cultured with TgCtwh3 or RH tachyzoites had an apparent decline, and correspondingly, the expression levels of those related proteins were notably decreased.

**Conclusions:**

Our findings suggest that TgCtwh3 may induce the apoptosis of the C17.2 cells by up-regulation of caspase-12, CHOP, and p-JNK, which are associated with ERS signaling pathways. This work contributes to better understanding the possible mechanism of brain pathology induced by *T. gondii* Chinese 1 isolates prevalent in China, and also reveals the potential value of ERS inhibitors to treat such related diseases in the future.

## Background

*Toxoplasma gondii* is a ubiquitous obligate intracellular protozoan parasite that can invade almost all nucleated cells from a wide range of warm-blooded animals [1]. It is estimated that up to one-third of the human population worldwide is chronically infected with this parasite [1,2]. In early molecular genotyping studies, *T. gondii* isolates in North America and Europe were classified into three genetic types (I, II, or III) [3-6], which vary substantially in virulence [7]. Type I strains are highly virulent to mice with an LD_100_ as low as a single parasite, while strains of types II and III are less virulent (LD_100_ > 1000) [8,9]. Recent population studies revealed that a few major clonal lineages of *T. gondii* dominate in different geographical regions, and the distribution of its genotype varies across the continents. The type II and III lineages are widespread in all continents, and dominate in Europe, Africa and NorthAmerica [10,11]. The type 12 lineage is the most common type in wildlife in North America [12,13], and the Africa 1 and 3 are among the major types in Africa [10,11,14,15]. Unlike in North America, *T. gondii* in Central and South America displays a highly diverse population structure, in which no genotype appears to be clearly dominant. Chinese 1, however, has been reported to be the most common type in East Asia, especially in China [16-20].

Significant differences in the host response to different *T. gondii* strains have been demonstrated in many previous studies. The secreted rhoptry kinase, ROP16, from types I and III, but not from type II, is involved in constitutive activation of the STAT transcription factors responsible for the alternative activation of macrophages [21,22], while the secreted dense granule protein, GRA15, from type II rather than types I and III, mediates high levels of NF-κB activation, promoting the classical activation of macrophages [23-25]. A strain-dependent host response was also found in murine microglial cells [26], chicken embryonic fibroblasts [27], and human neuroepithelial cells [28].

Most of the data currently available on *Toxoplasma*-host cell interactions were obtained using the three archetypes I, II, and III; however, the interaction of the atypical strains with the host cells remains unknown. It is important to determine how they differ from the canonical strains in modulating the host cell, because many reports showed that some atypical strains are correlated with more severe disease manifestations [24]. TgCtwh3, a representative strain of Chinese 1 that is predominantly prevalent in China, was found to have the polymorphisms of type I ROP16_I/III_ and type II GRA15_II_ in our preliminary effectors sequencing analysis [29]. The features of ROP16_I/III_ and GRA15_II_ of TgCtwh3 suggest that it may elicit a host response which is different from the archetypical stains of *Toxoplasma*. As a neurotropic parasite, *T. gondii* has a strong affinity for the central nervous system, resulting in encephalitis, intracranial calcifications, hydrocephalus, etc. [30]. Our previous study revealed that the canonical type I RH strain can induce apoptosis of the neural stem cells (NSCs) through endoplasmic reticulum stress (ERS) signaling pathways [31]. To identify the signaling pathways in neural stem cells uniquely modulated by TgCtwh3, a peculiar genotype with the characteristic of canonical type I and type II, we established the co-culture system using TgCtwh3 and the neural stem cell line C17.2 to detect the apoptosis level and the expression of apoptosis-related proteins.

## Methods

### Ethical statement

All animal experiments were conducted in strict accordance with the Chinese National Institute of Health Guide for the Care and Use of Laboratory Animals and approved by the Institutional Review Board of Anhui Medical University Institute of Biomedicine (Permit Number: AMU26-080610). All efforts were made to minimize animal suffering during all operational processes.

### Parasite

The tachyzoites of the mouse-virulent RH strain (type I), TgCtwh3 (Chinese I), and GFP-RH strain (GFP-labeled RH strain) were harvested from the mouse peritoneal exudates on day 3 or day 5 after infection, and were then isolated by centrifugation at 350 × g for 5 min to discard the contaminating host cells. After the supernatant was centrifuged at 1000 × g for 10 min, the parasites were washed twice and maintained by serial passage in the human foreskin fibroblasts (HFFs) monolayer for further experiments *in vitro*.

### Cell culture

The C17.2 murine neural stem cell line was developed and donated by Dr. Evan Y. Snyder of the Burnham Institute for Medical Research (La Jolla, CA, USA). The cell line was cultured, as described previously [32,33]. Briefly, the cells were seeded at a density of 5 × 10^4^ cells/cm^2^ in 75 cm^2^ flasks using 10 ml of Dulbecco’s Modified Eagle Medium (DMEM; Gibco, USA), supplemented with 10% (v/v) fetal bovine serum (Gibco, USA), 5% (v/v) horse serum (Gibco, USA), 2 mM l-glutamine (Gibco, USA), 100 U/ml penicillin (Sigma-Aldrich, USA) and 100 μg/ml streptomycin (Sigma-Aldrich, USA). The cells were incubated at 37°C in 5% CO_2_. The medium was replaced every 2–3 days. When the cell monolayer reached 70-80% confluence, the cells were detached with a solution of 0.05% trypsin-EDTA and reseeded.

### Identification of C17.2 using immunofluorescence

C17.2 cells were seeded on cover slips and cultured at 37°C in 5% CO_2_ for 24 h, then washed three times with PBS. Cells were fixed with 4% formaldehyde in PBS for 20 min at room temperature, washed with PBS, permeabilized with 0.3% Triton X-100 for 15 min, and then blocked with 1% bovine serum albumin in PBS for 1 h. After they were incubated with rabbit anti-Nestin monoclonal antibody (1:100; Sigma-Aldrich, USA) overnight at 4°C, the cover slips were balanced at 37°C for 1 h, washed three times with PBS, and incubated with FITC-conjugated goat anti-rabbit IgG (1:200; Santa Cruz, USA) for 60 minutes at 37°C. To observe the nucleus, cells were stained with Hoechst 33258 for 15 min at room temperature. Photographs were taken under a fluorescence microscope (Olympus, Japan).

### Establishment of C17.2 and *T. gondii* co-culture system and treatments

A co-culture system was established using transwell inserts (Corning, USA), as described previously, with minor modification [31]. Briefly, the bottom of the inserts is composed of polyester materials with a pore size of 0.4 μm, which only permits the permeabilization of small and soluble factors, but not *T. gondii* tachyzoites. The permeability of the co-culture system was first verified by adding GFP-RH tachyzoites to the upper chamber, and then the green fluorescent tachyzoites were monitored in the upper and lower chambers. For the co-culture treatments, two milliliters of single cell suspension of the C17.2 cells at a density of 1 × 10^5^/ml was seeded in the lower chamber of each insert. One milliliter of TgCtwh3 or RH strain tachyzoites with various doses (2 × 10^4^/ml, 1 × 10^5^/ml, or 5 × 10^5^ /ml) was added to the upper chamber of the inserts. The co-culture system was maintained in a basic culture medium supplemented with 10% fetal bovine serum and 5% horse serum (FBS; Gibco, USA) in 5% CO_2_ at 37°C. Apopida (apoptosis inducer A; Beyotime, China) was added to the upper chamber with a dilution of 1:3000 to be used as a positive control of apoptosis. As heat-inactivation controls, tachyzoites of TgCtwh3 or RH were heated with 100°C for 10 min and then added to the upper chamber. After co-culturing for 6 h, 12 h, and 24 h, the C17.2 cells were collected and the apoptotic levels were detected by flow cytometry (FCM).

To investigate the activity of the ERS pathway, the C17.2 cells were pretreated with 4 μmol/ L Z-ATAD-FMK (Biovision Inc., USA) for 6 h, and then 5 × 10^5^ TgCtwh3 or RH tachyzoites were added to the upper chamber. After 24 h incubation, the C17.2 cells were collected, the apoptotic level was detected by FCM, and the expression levels of CHOP, caspase-12, JNK, and p-JNK were detected using Western blotting.

### Detection of apoptosis

The apoptotic levels of the C17.2 cells were determined following the instruction of isothiocyanate (FITC)-annexin V/propidium iodide (PI) kit (BestBio, China). Briefly, cells were harvested, washed twice with cold PBS, and resuspended in 200 μl binding buffer (10 mM HEPES, 0.14 M NaCl, and 0.25 mM CaCl_2_). 5 μl FITC-conjugated annexin V was then added to the suspension and incubated at 4°C for 15 min in the dark. Next, 10 μl propidium iodide (PI) was added and incubated at 4°C for 5 min in the dark. Finally, 400 μL of 1 × binding buffer was added to each tube. The cells were analyzed using a flow cytometer (BD FACSCalibur, USA), and the data were analyzed using FCS Express 4.0 software. Annexin V-FITC^+^/PI^−^ cells represent the early apoptotic cells, and annexinV- FITC^+^/PI^+^ cells reflect the late apoptotic cells [34].

### Western blotting analysis

To further identify the apoptosis of C17.2 cells, the expression levels of caspase-3, CHOP, caspase-12, p-JNK, and JNK were determined using western blotting analysis. Western blotting was conducted as described previously [31]. Briefly, after the C17.2 cells were co-cultured with 5 × 10^5^ TgCtwh3 or RH tachyzoites for 24 h, they were harvested, washed with cold PBS, and lysed in a lysis buffer, including 50 mM Tris (pH 7.4), 150 mM NaCl, 1% Triton X-100, 1% sodium deoxycholate, and 0.1% SDS, supplemented with protease inhibitors and 1 mM phenylmethanesulfonyl fluoride (PMSF). The lysates were centrifuged at 12000 rpm for 10 min at 4°C and supernatants were collected. Protein concentrations in the supernatants were then measured using a BCA protein assay kit (Beyotime, China). Equal amounts of protein (20 μg for each sample) were separated on SDS-PAGE, and then electro-transferred onto a nitrocellulose membrane (Millipore, USA). After being rinsed in a TBST solution and blocked in 5% non-fat milk, the membranes were subsequently probed with the antibodies of caspase-3 (1:1000; Cell Signaling Technology, USA), CHOP (1:1000; Cell Signaling Technology, USA), caspase-12 (1:1000; Cell Signaling Technology, USA), phospho-c-Jun N-terminal kinase (p-JNK, 1:1000; Cell Signaling Technology, USA), c-Jun N-terminal kinase (JNK, 1:1000; Cell Signaling Technology, USA) or β-actin (1:1000; Cell Signaling Technology, USA) overnight at 4°C, and then incubated with a horseradish peroxidase-conjugated secondary antibody (1:5000; ZSGB-Bio, China) for 2 h at room temperature after being rinsed. Chemiluminescence was detected using an ECL kit (SuperSignal West Pico; Thermo Scientific, USA). The results were analyzed using Image J software (version 1.44).

### Statistical analysis

All quantitative data were expressed as mean ± SD. Student’s *t* test was used to analyze the statistical differences between two groups. Differences were considered statistically significant at a *P* value of <0.05.

## Results

### Verification of the co-culture system

**Figure 1:**
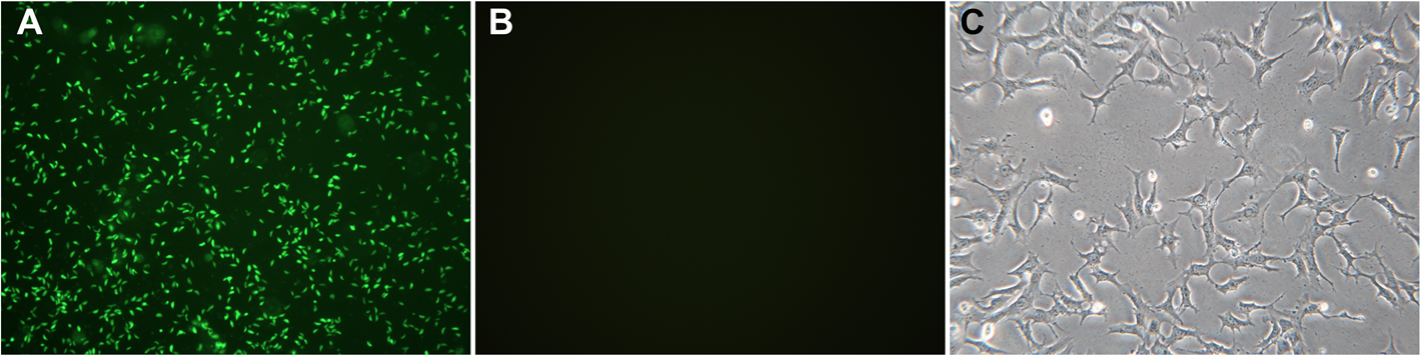
**Detection of GFP-RH tachyzoites in co-culture system. (A)** Fluorescent image of the upper chamber (×400); **(B)** fluorescent image of lower chamber (×400); **(C)** bright field image of the C17.2 cells in the lower chamber (×200).

To verify the permeability of the co-culture system, GFP-RH tachyzoites were added to the upper chamber. A large number of green fluorescent tachyzoites were found in the upper chamber under the fluorescence microscope, while no fluorescence was observed in the lower chamber (Figure 1). This indicates that *Toxoplasma* tachyzoites could not penetrate the filtration membrane into the lower chamber, and only excreted-secreted antigens (ESAs) of the parasite leaked into the lower chamber to exert effects on the C17.2 cells.

### TgCtwh3-induced apoptosis of C17.2 cells in time- and dose-dependent manners

**Figure 2:**
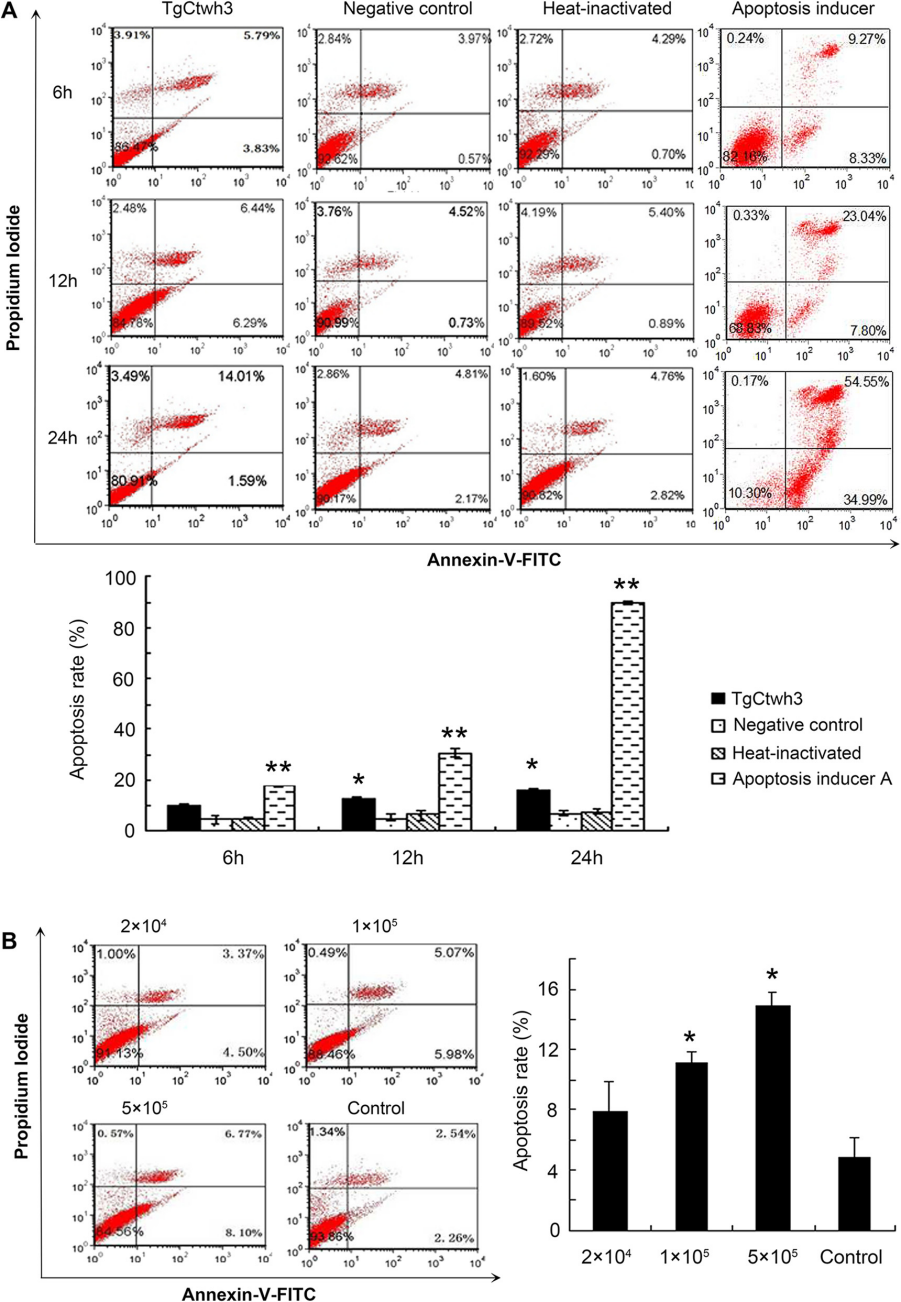
**Apoptosis detected by flow cytometry. (A)** The C17.2 cells were co-cultured with 5 × 10^5^ TgCtwh3 tachyzoites, heat-inactivated tachyzoites or Apopida (apoptosis inducer A) for 6 h, 12 h, and 24 h; **(B)** The C17.2 cells were co-cultured with various doses of TgCtwh3 tachyzoites (2 × 10^4^, 1 × 10^5^ and 5 × 10^5^) for 24 h. The cells were then collected, stained with Annexin V/PI, and analyzed by FCM. The plots are from a representative measurement and the graphics represent the mean and S.D. on three different assays (n = 3). ^*^ *P* < 0.05; ^**^ *P* < 0.01 vs. Negative controls.

After the co-culture with 1 × 10^5^ TgCtwh3 tachyzoites for 6 h, 12 h, and 24 h, the apoptosis rate of the C17.2 cells were 9.62 ± 1.02%, 12.73 ± 0.99%, and 15.6 ± 1.35%, while the apoptosis levels in the negative controls were 4.54 ± 1.72%, 5.25 ± 1.27%, and 6.98 ± 1.11%, respectively. The apoptosis rates of the heat-inactivation group were 4.99 ± 0.54%, 6.29 ± 2.08%, and 7.58 ± 1.16%, respectively. Significant differences were found among the tachyzoites co-culture group, the negative control group, and the heat-inactivation group (*P* < 0.05). No significant differences were found between the heat-inactivation group and the negative control group (Figure 2A).

**Figure 3:**
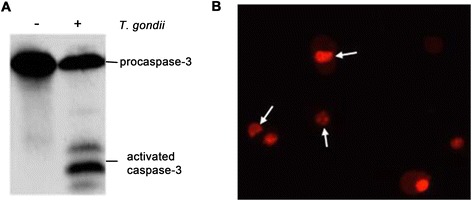
**Apoptosis detected by Western blotting and nucleus staining. (A)** The C17.2 cells were collected after they were co-cultured with 5 × 10^5^ RH tachyzoites for 24 h, and the activity of caspase-3 was detected by Western blotting; **(B)** The nuclei of the apoptotic cells were stained by propidium iodide. The arrows represent a shrinking nucleus or apoptotic body.

After the co-culture with 2 × 10^4^, 1 × 10^5^, 5 × 10^5^ TgCtwh3 tachyzoites for 24 h, the apoptosis rates of the C17.2 cells were 7.87 ± 2.01%, 11.05 ± 0.77%, and 14.87 ± 0.96%, respectively, and statistical differences were found when they were compared to the control group of 4.8 ± 1.32% (*P* < 0.05) (Figure 2B). Western blotting analysis revealed that caspase-3 of the *Toxoplasma* co-culture group was activated (Figure 3A). Nuclear staining showed that the nuclei of the C17.2 cells co-cultured with the *Toxoplasma* tachyzoites began shrinking; some nuclei appeared to have an apoptotic body (Figure 3B), which further verified the cell apoptosis.

### TgCtwh3 induced apoptosis of C17.2 cells via ERS pathway

**Figure 4:**
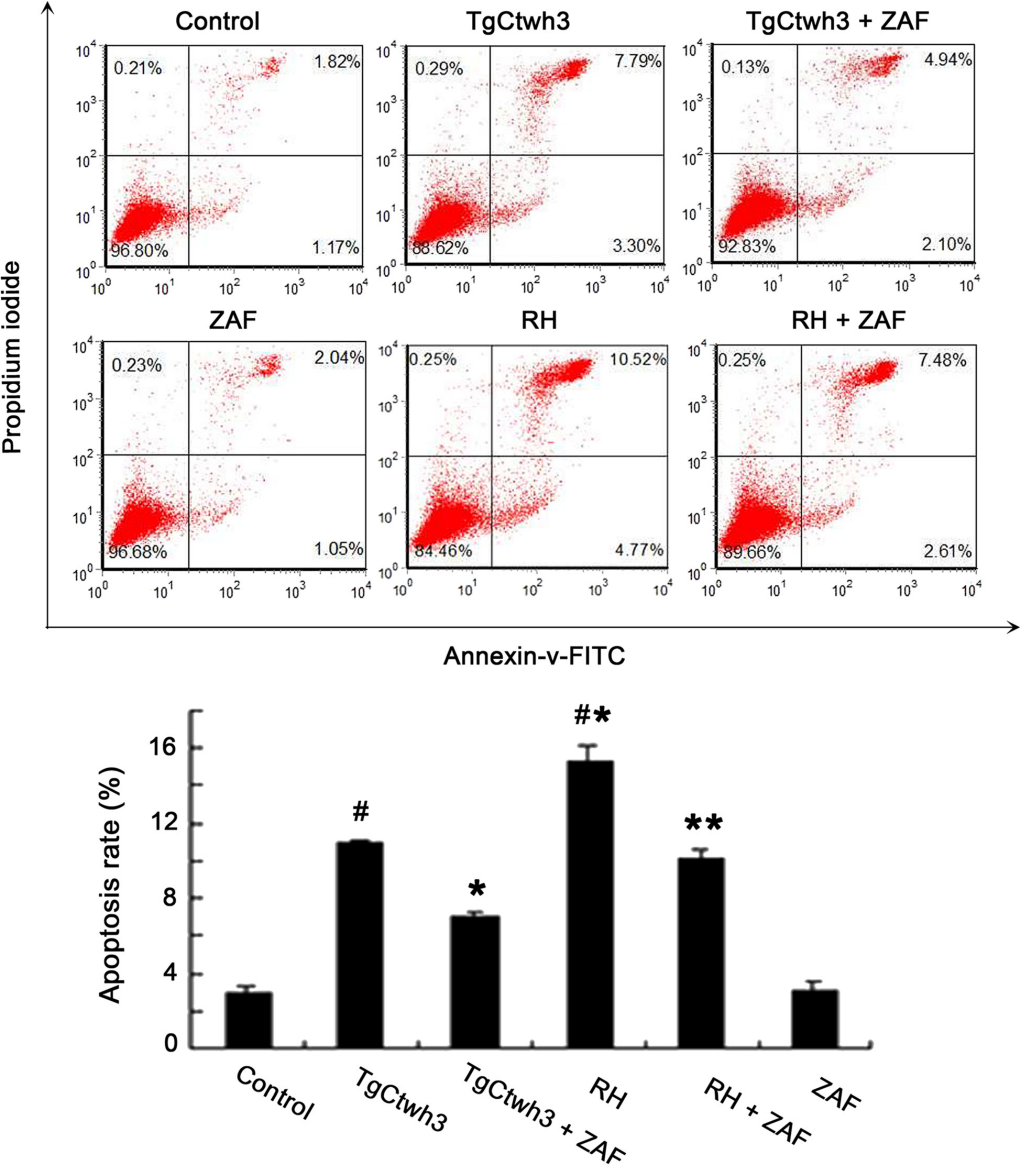
**Effect of inhibitors on the apoptosis levels of C17.2 cells.** After the C17.2 cells were pretreated with or without Z-ATAD-FMK (ZAF) for 6 h, they were co-cultured with 5 × 10^5^ TgCtwh3 or RH tachyzoites for 24 h. The cells were then collected, stained with Annexin V/PI, and analyzed by FCM. TgCtwh3 or RH + ZAF stands for the C17.2 cells pretreated with Z-ATAD-FMK, and then co-cultured with either TgCtwh3 or RH tachyzoites. The data are from three independent experiments. ^*^ *P* < 0.05 vs. TgCtwh3 co-cultured group; ^**^ *P* < 0.05 vs. RH co-cultured group; ^#^ *P* < 0.05 vs. control.

To examine whether an ERS pathway was involved in the apoptosis of the C17.2 cells induced by TgCtwh3, the cells were pretreated with 4 μmol/L Z-ATAD-FMK for 6 h before 5 × 10^5^ TgCtwh3 or RH tachyzoites were added to the upper chamber. After the pretreatment of Z-ATAD-FMK, the apoptosis rates of the C17.2 cells co-cultured with TgCtwh3 tachyzoites or RH tachyzoites were 7.04 ± 0.33% and 10.09 ± 0.52%, significantly lower than those in the Z-ATAD-FMK untreated TgCtwh3 group (11.09 ± 0.09%) and untreated RH group (15.29 ± 0.84%), accordingly (*P* < 0.05) (Figure 4). The apoptosis rates of the C17.2 cells co-cultured with TgCtwh3 (11.09 ± 0.09%) were significantly lower than RH co-culture group (15.29 ± 0.84%) (Figure 4).

**Figure 5:**
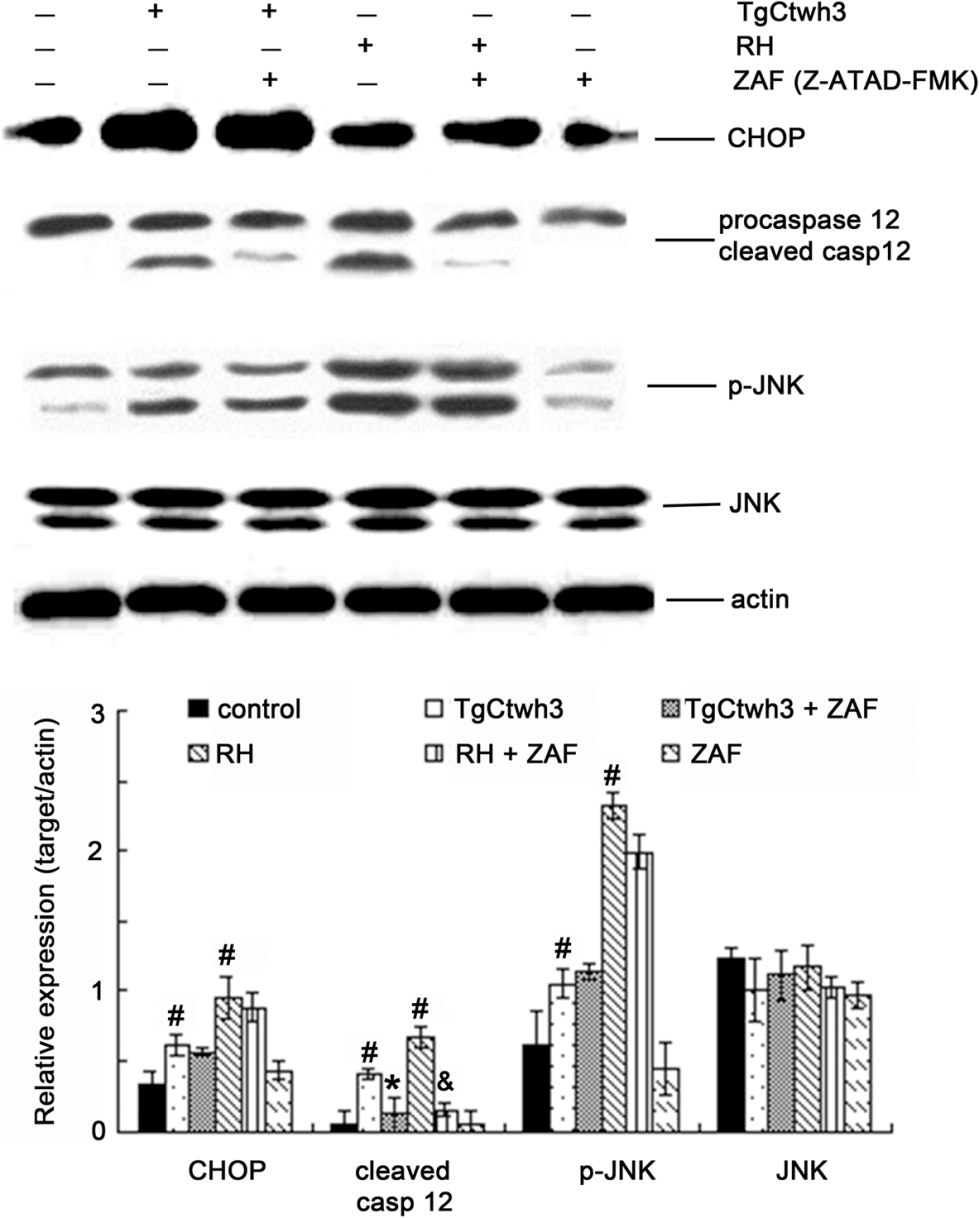
**Expression levels of caspase-12, JNK, and CHOP in C17.2 cells.** After the C17.2 cells were pretreated with or without Z-ATAD-FMK (ZAF), they were co-cultured with 5 × 10^5^ TgCtwh3 or RH tachyzoites for 24 h. The presented figures are from a representative study and the graphics represent the mean and SD on different assays (n = 3). The C17.2 cells without the co-culture of tachyzoites or only pretreated with ZAF were used as controls. TgCtwh3 or RH + ZAF stands for the C17.2 cells pretreated with Z-ATAD-FMK, and then co-cultured with either TgCtwh3 or RH tachyzoites. The experiments were repeated three times. ^#^ *P* < 0.05 vs. control; *P < 0.05 vs. TgCtwh3 co-cultured group; & *P* < 0.05 vs. RH co-cultured group.

Western blotting analysis further displays that the expression levels of cleaved caspase-12, CHOP, and p-JNK in the C17.2 cells increased significantly in both the TgCtwh3 and RH tachyzoites co-cultured groups when compared to the control group. When the C17.2 cells were pretreated with a caspase-12 inhibitor, Z-ATAD-FMK, the expression level of cleaved caspase-12 in the inhibitor-pretreated groups significantly decreased (*P* < 0.05), whereas no significant differences were detected in the levels of CHOP and p-JNK (Figure 5) when they were compared to TgCtwh3 or RH tachyzoites co-cultured groups.

## Discussion

Recent research revealed that *T. gondii* appears to have a limited genetic diversity in Asia, particularly in China. By using both PCR-RFLP and microsatellite typing, the majority of strains isolated from animals, and humans as well, belong to one lineage corresponding to the previously described ToxoDB#9, termed as Chinese 1 [11,35]. TgCtwh3 is a representative strain of Chinese 1, which was found to have high virulence to mice as type I RH strain [16,36]. Our previous studies revealed that TgCtwh3 shares the polymorphism of the kinase domain of ROP16_I/III_ at 503 L with that of GT1 strain (type I), which is necessary to sustain STAT3 phosphorylation, and subsequently drive macrophages to M2 cells polarization. Additionally, TgCtwh3 has a polymorphic feature of GRA15_II_ (type II), which is responsible for M1 cells polarization. It is postulated that TgCtwh3 may induce the distinct host response and pathogenesis of toxoplasmosis, for example, toxoplasmic encephalopathy.

Apoptosis is a programmed cell death that is activated by several stimuli, including intracellular parasites. A loss of control of cell death (resulting in excess apoptosis) is involved in the pathogenesis of many infectious diseases, leading to neurodegenerative diseases, hematologic diseases, and tissue damage [37-39]. *T. gondii* is able to promote or inhibit the cell apoptotic machinery, depending on the host cell type, infection stage as well as its virulence and parasite load [40]. Neural stem cells (NSCs) are self-renewing, multipotent cells that generate the main phenotype of the nervous system and play an important role in the development of the central nervous system. Previous research reported that abnormal apoptosis of the NSCs induced by congenital infection of viruses could lead to brain malformations [41-43]. Few studies have examined the effect of *T. gondii* infection on the NSCs [31,44], in which only RH (type I) was used. In the present study, we investigated the effects of the atypical Chinese 1 strain TgCtwh3 on the neural stem cell line C17.2. The results show that TgCtwh3 induced apoptosis as early as 12 h after co-culture with the C17.2 cells in a dose-dependent manner (Figure 2). This differs from our previous experiments with primary NSCs of embryos of ICR mice, that showed a significant apoptosis at 24 h after infection with RH tachyzoites [31]. The discrepancy might be related to different Toxoplasma genotypes and different host cell types. Additionally, we found that, after the co-culture with the parasites for 24 h, the apoptosis level of C17.2 cells induced by TgCtwh3 was significantly lower than that induced by RH. The possible involvement of the background of ROP_I/III_ and GRA15_II_ effectors in the Chinese 1 strain remains to be elucidated.

To date, two methods are widely used to prepare ESAs of *T. gondii*. The first is to prepare the ESAs from peritoneal fluids of mice intraperitoneally infected with tachyzoites, and another is to harvest the ESAs from the supernatant of cultured tachyzoites *in vitro* [45-48]. Either may contain unknown components and result in an unexpected effect on the NSCs in the following experiments. Therefore, the co-culture system of the C17.2 cells and tachzoites, instead of excreted-secreted antigens (ESAs), was directly used in the present study. To test the effectiveness of this barrier system, GFP-RH tachyzoites were added to the upper chamber, and no GFP–RH tachyzoites were found in the lower chamber (Figure 1), indicating that the apoptosis of the C17.2 cells was undoubtedly induced by the ESAs of the parasite rather than the direct interaction of tachyzoites. It has been found that the major host cells could act as bystanders in acute infection, and apoptosis of the bystander host cells may result from the secretion of some soluble factors by parasite-infected cells [49,50]. The composition of the *T. gondii* ESAs is surprisingly complex, and only few microneme proteins, rhoptry proteins, and dense granule proteins have been identified [46,51]. ROP18, a major roptry effector molecule, targets the host endoplasmic reticulum-bound transcription factor ATF6beta which is involved in the apoptosis through ERS pathway [52]. The functional role of ROP18 in the apoptosis of the C17.2 cells induced by TgCtwh3 is under investigation.

It has been reported that apoptosis mediated by ERS pathway plays a key role in many diseases [37-39,53,54]. Our preliminary study on mouse gene expression profiling in primary NSCs infected by the RH strain indicates that the expression of some important genes involved in the ERS-induced apoptosis signal pathway were remarkably altered. To date, three apoptotic pathways triggered by ER stress have been reported. The first is the transcriptional induction of the gene for CHOP (C/EBP homologous protein)/GADD153, which is barely detected under physiological conditions, but is strongly induced in response to ER stress [55,56]. The second is the activation of the cJUN NH2-terminal kinase (JNK) pathway [57]. The third is the activation of the caspase-12, which is activated by ER stress, but apparently not by death receptor-mediated or mitochondria -targeted apoptotic signals [58,59]. In the present study, we detected the expression levels of related proteins involved in the ERS-mediated apoptosis pathway in the C17.2 cells/ *Toxoplasma* co-culture system. Western blotting analyses show that the co-culture of both TgCtwh3 and RH strains up-regulated the expression levels of CHOP, activated caspase-12, and p-JNK in the C17.2 cells. Meanwhile, the caspase-12 inhibitor Z-ATAD-FMK reduced the apoptosis induced by TgCtwh3. These results were consistent with our previous data found in primary NSCs infected by RH tachyzoites, indicating that both TgCtwh3 and RH strains induced the apoptosis of NSC cell line or primary NSCs through the ERS pathway.

## Conclusions

The ESAs of TgCtwh3, a representative strain of type Chinese 1 mostly found in China, induced the apoptosis of the C17.2 cells as early as 12 h after co-culture. The ESAs derived from both TgCtwh3 and RH induced C17.2 cells apoptosis via ERS signaling pathways but TgCtwh3 is a lower inducer than the RH strain. Our findings contribute to better understanding the possible mechanism of brain pathology caused by *T. gondii* prevalent in China, and also reveal the potential value of ERS inhibitors in treatment strategy of *Toxoplasmic* encephalopathy.
